# Expanding our view of *Bartonella* and its hosts: *Bartonella* in nest ectoparasites and their migratory avian hosts

**DOI:** 10.1186/s13071-020-3896-7

**Published:** 2020-01-10

**Authors:** Heather M. Williams, Katharina Dittmar

**Affiliations:** 1 Department of Environment and Sustainability, State University of New York at Buffalo, North Campus, 602 Clemens Hall, Buffalo, NY 14260 USA; 2 Department of Biological Sciences, State University of New York at Buffalo, North Campus, 109 Cooke Hall, Buffalo, NY 14260 USA

**Keywords:** *16S* rRNA, *Bartonella*, *Ceratophyllus*, *Dermanyssus*, Eastern bluebird (*Sialia sialis*), *gltA*, *Protocalliphora*, Purple martin (*Progne subis*), Tree swallow (*Tachycineta bicolor*)

## Abstract

**Background:**

*Bartonella* is a genus of Gram-negative facultative intracellular Alphaproteobacteria of public health importance. Although they are known to mainly infect mammalian hosts with some blood-feeding arthropods having been confirmed as vectors, there is some evidence of *Bartonella* association with non-mammalian hosts including birds.

**Methods:**

Here we used high-throughput sequencing of *16S* rRNA and Sanger sequencing of the citrate synthase (*gltA*) genes to test for the presence of *Bartonellaceae* in the blood of three migratory cavity nesting bird species, purple martins (*Progne subis*), tree swallows (*Tachycineta bicolor*) and eastern bluebirds (*Sialia sialis*) and their most prevalent and abundant nest ectoparasites, *Dermanyssus prognephilus* (mite), *Ceratophyllus idius* (flea) and *Protocalliphora sialia* (bird blow fly larva). We constructed maximum likelihood phylogenetic trees to verify the placement of the resulting sequences in the *Bartonellaceae*.

**Results:**

We found evidence of *Bartonella* in all three bird species and all three arthropod species tested. We report multiple instances of identical *Bartonella* sequences in both birds and parasites, leading to the likely hypothesis that these ectoparasites are potential vectors of *Bartonella*. Our phylogenetic analysis suggests that ‘avian *Bartonella*’ may form its own sub-clade within the genus *Bartonella*.

**Conclusions:**

To the best of our knowledge, we provide the first confirmation of overlapping *Bartonella* strains among bird hosts and various species of nest-associated ectoparasites from the same system, suggesting a possible *Bartonella* host–vector relationship between these arthropods and a non-mammalian host. Our study adds to the growing appreciation of the *Bartonellaceae* as a phylogenetically diverse group with a wide range of hosts.
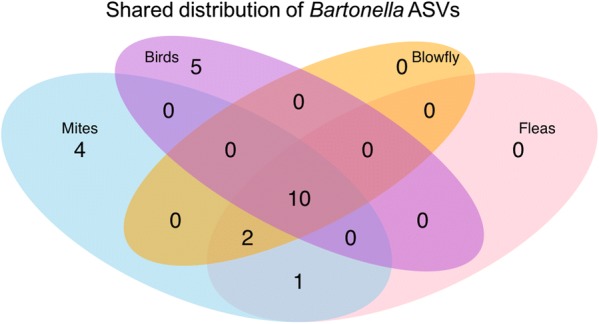

## Background

*Bartonella* is a genus of Gram-negative facultative intracellular Alphaproteobacteria whose niche is traditionally described as within the red-blood cells of mammalian hosts, from which it is transferred between hosts by blood-feeding arthropods [1, 2]. The genus comprised a single species (*Bartonella quintana*) until as recently as 1993, but since then increased diversity has been recognized with 35 species with confirmed nomenclature and a further 10 putative species now in the literature [3]. In parallel with the increasing recognition of diversity within *Bartonella*, there has also been an expanding list of known host species. Indeed, our knowledge of *Bartonella* is no longer strictly limited to mammal-arthropod systems, as it has now been isolated by two studies in non-mammalian vertebrate hosts. One study found it in loggerhead sea turtles (*Caretta caretta*) [4] and another study found *B. henselae* and *B. koehlerae* in blood samples from North American wild birds [northern mockingbirds (*Mimus polyglottos*), red-winged blackbirds (*Agelaius phoeniceus*), red-bellied woodpeckers (*Melanerpes carolinus*) and common loons (*Gavia immer*)] [5]. If *Bartonella* is circulating in wild birds, it may also be assumed to be in some subset of hematophagous avian ectoparasites. Accordingly, the presence of *B. grahamii* has been confirmed in the tick species *Ixodes turdus* which was collected from migratory birds in Korea [6] and in the hematophagous mites of the bird-associated genus *Dermanyssus* in the Czech Republic [7]. Furthermore, a recent study found a predominance of ‘*Bartonella*-like’ bacteria in the microbiome of the poultry ectoparasite, *Dermanyssus gallinae* [8]. Although this study did not achieve a high taxonomic resolution on these bacteria (leading the authors to conservatively label it as ‘*Bartonella*-like’ bacteria), this could be of high commercial and animal-health interest as *D. gallinae* has a worldwide distribution with over 80% of poultry farms reporting infestations [9].

Although evidence for *Bartonella* in avian host–parasite systems is clearly growing, there has not so far been any study to link presence of *Bartonella* between avian-associated arthropods (e.g. hematophagous ectoparasites) and birds. Cavity-nesting birds and especially those using man-made nest boxes [10] are likely candidates for providing a fitting model as they are associated with a high load of hematophagous nest ectoparasites [11, 12]. Three species which fit this profile are purple martins (*Progne subis*), eastern bluebirds (*Sialia sialis*) and tree swallows (*Tachycineta bicolor*). They are all wide-ranging migrant cavity-nesting birds, which are sympatric throughout much of their breeding ranges and regularly (or exclusively in the case of the eastern subspecies of the purple martin) make their nests in man-made nest boxes [13–15]. While the taxonomic composition of their nest parasites appears to vary throughout their ranges [13–16], at our field site in western New York State, three hematophagous ectoparasite species (a mite, *Dermanyssus prognephilus*; a flea, *Ceratophyllus idius*; and a bird blow fly larva, *Protocalliphora sialia*) are found at high prevalence (and generally high abundance) in the nests of all three bird species as well as on the birds (HMW, unpublished data).

Here we use high-throughput sequencing of the bacterial *16S* rRNA paired with Sanger sequencing of the citrate synthase (*gltA*) gene to detect *Bartonella* DNA in these three species of nest-associated ectoparasites and in the blood of nestlings of three of their avian host species. We discuss the phylogenetic placement of these sequences in terms of known *Bartonella* diversity and the potential for in-nest vector relationships under the hypothesis that overlapping sequences will be identified in both birds and their ectoparasites.

## Methods

### Sample collection

Fieldwork was carried out at Iroquois National Wildlife Refuge and Tonawanda Wildlife Management Area in Genesee County, New York State in the summers of 2017 and 2018. Purple martin, eastern bluebird and tree swallow nests were monitored as part of a wider project documenting the effect of nest-associated ectoparasites on nestling fitness. Blood samples were drawn from nestlings of 49 purple martins from 33 different nests; from 6 eastern bluebirds from 4 nests and from 6 tree swallows from 4 nests. The unequal sample size reflects the priorities of the aforementioned wider project. Nestlings were sampled at approximately 2/3 of their way through the nestling period (purple martin nestlings were aged between 16–20 days-old at the time of blood draw, with a mean fledging age of 28 days, whereas bluebird and tree swallow nestlings were sampled between 11–12 days-old and typically fledge at 18 and 20 days-old, respectively) to allow a sufficient period for a potential *Bartonella* infection to proliferate in the blood without risking prompting premature fledging by handling birds too late in their nestling stage [17]. We chose to sample nestlings rather than adults to reduce the number of possible of origins of a potential *Bartonella* infection. In each case blood was drawn from the brachial vein using a 27.5 gauge needle to collect a maximum of 140 µl of blood in heparinized microhematocrit capillary tubes. Blood samples were kept on ice in the field until being stored at − 70 °C on the same day until samples were processed.

Nest materials were collected after the birds fledged and were placed in Berlese funnels for 2–3 h to allow nest-dwelling invertebrates to collect in ethanol. Three hematophagous parasites were common in the sampled nests of all three bird species, the mite *Dermanyssus prognephilus,* the flea *Ceratophyllus idius* and the bird blow fly larva *Protocalliphora sialia*. Nest materials from purple martins were also sampled at regular intervals during the nesting period as part of a wider study. The same three species of nest-associated ectoparasites were commonly present at every sampling period. Parasites were identified *via* morphology following Moss [18], Traub et al. [19] and Sabrosky et al. [16], respectively, using a Zeiss microscope. Specimens were stored in 95% ethanol at − 70 °C and washed in PBS prior to DNA extraction. All samples (blood and ectoparasites) were collected between the last week in June and the first week in August in both years.

### DNA extraction

DNA was extracted from blood samples (*n* = 61) using the Qiagen DNeasy Blood and Tissue Kit (Qiagen, Valencia, CA, USA), directly following the handbook Spin-Column protocol for nucleated animal blood. In brief, 20 μl of proteinase K was added to 10 μl of blood, 190 μl of PBS and 200 μl of buffer AL and vortexed prior to incubation at 56 °C for 10 min. 200 μl of ethanol was added to the sample prior to centrifugation at 8000× *rpm* for 1 min in a spin column. Two further centrifugation steps were carried out, with 500 μl of buffer AW1 and then 500 μl of buffer AW2 added at each step. We used two elutions using 100 μl of buffer AE and 1 min of centrifugation at 8000× *rpm* before the DNA eluant was transferred to a stock tube.

Ectoparasite samples (*D. prognephilus*: *n* = 69; *C. idius*: *n* = 76; *P. sialia*: *n* = 12) were washed in PBS, then homogenized using sterile razor blades and incubated at 56 °C overnight in 180 μl ATL and 20 μl proteinase K. DNA was then extracted as per the Qiagen DNeasy Blood and Tissue Kit handbook protocol for Spin-Column animal tissues extractions. In summary, samples were vortexed and incubated for 1 h at 56 °C. We then added 200 μl of buffer AL and 200 μl of ethanol, transferred the solution to a spin column and centrifuged at 8000× *rpm* for 1 min. Two further centrifugation steps were carried out using buffer AW1 and buffer AW2 as was done for the blood samples. Finally, we used two elutions of 75 μl buffer AE rather than the standard 200 μl so as to increase the DNA concentration. Each sample contained multiple ectoparasite individuals of the same species from the same nest (8 for *D. prognephilus*, 4 for *C. idius* and 2 for *P. sialia*) to ensure sufficient DNA concentrations for analysis.

### *16S* rRNA microbiome sequencing

We sequenced the hypervariable V3–V4 region of bacterial *16S* rRNA from all samples as part of a separate microbiome study. PCR, library preparation and sequencing were carried out by the University at Buffalo Genomics and Bioinformatics Core facility following the *16S* metagenomics sequencing library preparation guide for the Illumina MiSeq system. Initial DNA concentrations were measured using a Qubit Fluorometer and were adjusted to 5 ng/μl. In the first stage PCR, 2.5 μl of DNA was mixed with 12.5 μl 2× KAPA HiFi HotStart Readymix and 5 μl each of 1 μM forward and reverse primers (forward: 5′-CCT ACG GGN GGC WGC AG-3′, reverse: 5′-GAC TAC HVG GGT ATC TAA TCC-3′). This primer set was chosen as it has been found to have the best overall coverage for this bacterial region [20]. PCR was performed in a thermal cycler at initial denaturation at 95 °C for 3 min, followed by 25 cycles of 95 °C for 30 s, 55 °C for 30 s and 72 °C for 30 s followed by a final elongation step for 5 min at 72 °C. Second stage PCR allowed for attachment of Illumina sequencing adapters. 5 μl of first stage PCR product was added to each well on a new plate with 5 μl of each index primer, 25 μl 2× KAPA HiFi HotStart Readymix and 10 μl of PCR-grade water. PCR was performed in a thermal cycler at initial denaturation at 95 °C for 3 min, followed by 8 cycles of 95 °C for 30 s, 55 °C for 30 s and 72 °C for 30 s followed by a final elongation step for 5 min at 72 °C. PCR clean-up was performed after each reaction using 20 μl of AMPure XP beads in each well, final concentrations were measured using a Quant IT DsDNA Assay Kit and adjusted to 4 nM using EB buffer. Libraries were denatured using NaOH, diluted with hybridization buffer and heat denatured. Samples were loaded with 5% PhiX internal controls and were sequenced using the Illumina Miseq with 300-cycle paired-end sequencing with a negative control (no DNA added) on each plate.

Raw sequences were imported to QIIME2 (https://qiime2.org) as paired-end sequences after primer sequence removal and demultiplexed. We used DADA2 [21] to remove chimeras and to denoise our sequences by trimming the first 5 bases from both the forward and reverse sequences and truncating the reverse sequences at 223 bases based on sequence quality. We trained a naïve Bayesian classifier using the Greengenes 99% OTU database from version 13.8 [22] and extracted reads from the reference database which match the sequencing primer pair. We then used the classifier to cluster our reads to 1% Amplicon Sequence Variants (ASVs) and assign taxonomy.

### *16S* rRNA *Bartonella* verification

In this microbiome study, 40 different ASVs identified to the family *Bartonellaceae* in our classification system, none of which occurred in the 4 negative controls (one per plate). We cross-referenced these sequences with NCBI GenBank using the BLASTn tool to perform a search limited to the *Bartonellaceae*. All 40 ASVs were associated with *Bartonella* sequences with low e-values and generally high coverage (Additional file 1: Table S1).

From GenBank we obtained a total of 93 homologous *16S* rRNA sequences including 33 *Bartonella* species and sister taxa including 10 species of *Mesorhizobium*, 10 species of *Ochrobactrum*, 12 species of *Brucella* and 1 species of *Rhizobium* to use as outgroup (Additional file 2: Table S2). We also included 10 sequences classified as ‘*Bartonella*-like’ derived from *Dermanyssus gallinae* [8] (a taxon related to our mite species, *D. prognephilus*) (Additional file 2: Table S2). We were not able to include comparison sequences from the only other study of avian *Bartonella* [5] or from the study of *Bartonella* in avian ticks [6] as they sequenced different genomic regions. Sequences were aligned in Seaview v.4.6.5 [23] using Clustal-O. The aligned sequences were trimmed to consistently include a 430 bp of the *16S* rRNA V3–V4 region across all samples.

To maintain a conservative analysis, we removed some ASVs on from our analysis at this point based on the following criteria: (i) two ASVs (Mite_2 and Mite_4) were removed due to a relatively poor alignment; (ii) four ASVs (Mite_8, Mite_9, Mite_10, Mite_12) were removed as they had shorter sequences than the other ASVs; (iii) Nine ASVs (Mite_7, Mite_14, Flea_1, Flea_3, Flea_4, Flea_5, PUMA_1, PUMA_3, TRES_2) were removed as there were fewer than 20 reads in the full dataset; and (iv) seven ASVs (Mite_1, Mite_5, Mite_6, Mite_11, Mite_16, Flea_2, PUMA_2) were removed as their percentage identity with the *Bartonellaceae* from BLASTn was in the lower quartile of our samples (i.e. below 98%). After quality filtering, we were left with 22 ASVs which were submitted to NCBI GenBank: MN320509-MN320530.

We used MEGA7 [24] to find the best-fit nucleotide substitution model for our data according to BICc. As K2 + G + I was selected as the best-fit model (Additional file 3: Table S3), we created a maximum likelihood tree in Seaview using the K80 substitution model, optimized invariable sites (I) and optimized across site rate variation (G), with 100 bootstrap replicates and the best of nearest neighbor and subtree pruning with 5 random starts. The resulting tree was exported from Seaview to FigTree v.1.4.4 [http://tree.bio.ed.ac.uk/software/figtree/] for editing.

### PCR confirmation of *Bartonella gltA*

While the *16S* rRNA gene is an appropriate target for metagenomics studies, it does not always provide high resolution for *Bartonella* sequences [25]. To support our identification of *Bartonella*, we therefore performed PCRs for the *Bartonella* citrate synthase gene (*gltA*) for a representative subset of our samples, which contained *16S* rRNA *Bartonella* sequences. Two samples from eastern bluebird blood, 2 samples from tree swallow blood, 9 samples from purple martin blood, 7 samples from *C. idius* and 13 samples of *D. prognephilus* were selected for analysis.

PCR amplifications were performed in a 25 μl reaction including 1 μl of DNA, 0.2 μl of 5 U/μl Taq, 0.5 μl each of 10 μM forward and reverse primers, 2.5 μl of 50 mM dNTPs, 2.5 μl of 10× buffer, 1.5 μl of MgSO_4_ and 13.8 μl of PCR grade water. We used BhCS781.p (5′-GGG GAC CAG CTC ATG GTG G-3′) and BhCS1137.n (5′-AAT GCA AAA AGA ACA GTA AAC A-3′) primers to generate a 379-bp amplicon of the *Bartonella gltA* gene [26]. PCR reactions were performed with the following cycling conditions: one 5-min cycle at 95 °C, followed by 30 cycles of 95 °C for 30 s, 50 °C for 30 s and 72 °C for 40 s, followed by 1 final cycle at 72 °C for 7 min. PCR clean-up was performed using AMPure XP beads. Positive amplicons were sequenced with an ABI 3730XL sequencer at TACGen (http://www.tacgen.com). PCR-positive bands were obtained in all *D. prognephilus* samples (*n* = 13), all but 2 of the *C. idius* samples (*n* = 8) and all but 2 of the avian blood samples (*n* = 13). Paired-end sequences were merged, and the quality of the resulting consensus sequences was assessed from chromatograms in Geneious v.8.0.5 (https://www.geneious.com). However, sequence quality was very poor for all blood and *C. idius* samples and three of the *D. prognephilus* samples and these samples conservatively recorded as negative for *Bartonella* and excluded from further analysis. All of the 10 mite samples with high quality consensus *gltA* sequences were identified to *Bartonella* sequences using a BLASTn search (Additional file 1: Table S1). These 10 ASVs were submitted to NCBI GenBank: MN371264–MN371273.

As individual samples generated multiple *Bartonella* ASVs in the *16S* rRNA sequencing, we did not concatenate our sequences, but rather generated a second phylogeny based on the *gltA* data to compare with the *16S* rRNA tree. To that end we downloaded 51 representative homologous sequences including 13 species of *Bartonella*, 2 species of *Mesorhizobium* along with 7 species of *Brucella* sequences from GenBank, along with 3 species of *Rhizobium* to use as the outgroup (Additional file 2: Table S2). Sequences were aligned in Seaview using the Clustal-O algorithm and trimmed to the 379 bp region of interest. MEGA7 indicated that K2 + G was the best substitution model for the data (Additional file 4: Table S4), so Seaview was used with the K80 model and optimized across site rate variation (G), with 100 bootstrap replicates and the best of nearest neighbor and subtree pruning with 5 random starts. The resulting tree was exported from Seaview to FigTree for editing.

## Results

### *Bartonella* sequences from *16S* rRNA sequencing

Sequences assigned to *Bartonella* were found in all three nest-associated ectoparasite species and all three cavity-nesting bird species (Additional file 5: Table S5). Prevalence of *Bartonella* in the bird species ranged between 33% (2/6) for eastern bluebirds, 39% (19/49) and 83% (5/6) for tree swallows. Ectoparasite samples were comprised of multiple individuals (8 for *D. prognephilus*, 4 for *C. idius* and 2 for *P. sialia*) which complicates the calculation of prevalence. At the sample level, a quarter of the *P. sialia* samples (3/12) and 36% of the *C. idius* samples (27/76) were *Bartonella*-positive. Only one *D. prognephilus* sample out of 57 did not contain *Bartonella*, but as that sample had a low total number of reads compared with other samples, we consider prevalence to approach 100%. Considering the limits of individual prevalence, these ranged between 13–25% in *P. sialia*, 9–36% in *C. idius* and 13–100% in *D. prognephilus*. The mites had a very high relative abundance of *Bartonella* sequences in their microbiome compared with the other arthropod and avian species (Table 1). Every nest we sampled contained some evidence of *Bartonella*, whether that be in the ectoparasites, in the birds, or (typically) both (Additional file 5: Table S5).

**Table 1:** Relative abundance of the *Bartonellaceae* in the *16S* rRNA microbiome of studied host species

Host species	Minimum (%)	Maximum (%)	Mean (%)
*Dermanyssus prognephilus*	1.55	98.57	18.45
*Ceratophyllus idius*	0	25	0.91
*Protocalliphora sialia*	0	1.13	0.13
Purple martin (*Progne subis*)	0	1.04	0.13
Tree swallow (*Tachycineta bicolor*)	0	0.50	0.22
Eastern bluebird (*Sialia sialis*)	0	0.13	0.04

*Notes*: The mite, *Dermanyssus prognephilus* has a very high relative abundance of *Bartonella* compared with the other species. Relative abundances for *D. prognephilus* are calculated after the exclusion of one sample with a low number of reads (see text)

**Fig. 1:**
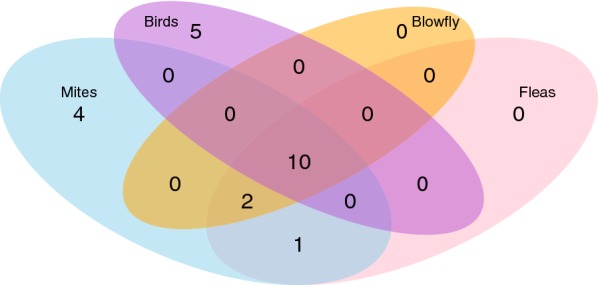
Venn diagram showing distribution of unique *16S* rRNA *Bartonella* ASVs between the three ectoparasite hosts [mites (*Dermanyssus prognephilus*), blow fly (*Protocalliphora sialia*) and fleas *Ceratophyllus idius*] and the three bird species tested [purple martins (*Progne subis*), tree swallows (*Tachycineta bicolor*) and eastern bluebirds (*Sialia sialis*)]. Ten *Bartonella* ASVs were found in all host species tested

Multiple ASVs of *Bartonella* were found in all species tested; many individual birds harbored multiple *Bartonella* ASVs and many nests contained ectoparasite species containing multiple *Bartonella* sequences (Table 2). Of the 22 unique sequences of *Bartonella* found in our dataset after quality filtering, 5 ASVs were found only in birds and 7 ASVs were only found in nest-associated ectoparasites. Of particular interest, however, are the 10 strains which were isolated in both birds and ectoparasites (Fig. 1). Three of these cross-taxa strains included examples of the same *Bartonella* sequence existing in the nestlings and parasites from the same nest, providing strong support for a likely vector–host relationship.

**Table 2:** Distribution of unique *Bartonella* ASVs from *16S* rRNA sequencing of nestling blood samples and nest-associated ectoparasite microbiomes

Host species	No. of unique *Bartonella* ASVs per host species (*16S* rRNA)	Maximum no. of *Bartonella* ASVs per sample or individual (*16S* rRNA)
*Dermanyssus prognephilus*	17	8
*Ceratophyllus idius*	13	7
*Protocalliphora sialia*	12	4
Purple martin (*Progne subis*)	10	10
Tree swallow (*Tachycineta bicolor*)	8	8
Eastern bluebird (*Sialia sialis*)	2	1

**Fig. 2:**
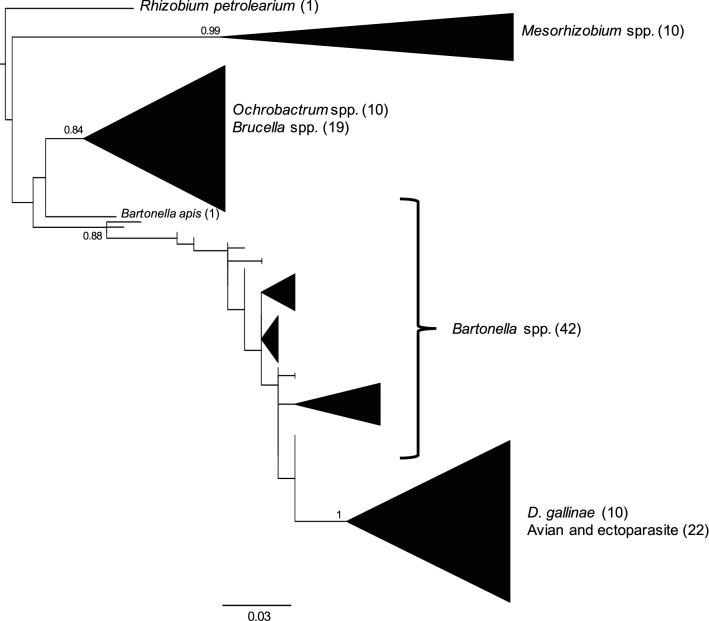
Maximum likelihood phylogenetic tree showing the placement of our *16S* rRNA *Bartonella* sequences within a wider taxonomy. The tree used the K80 substitution model, optimized invariable sites (I) and optimized across site rate variation (G), with 100 bootstrap replicates and the best of nearest neighbor and subtree pruning with 5 random starts. Numbers on branches show bootstrap support values. Only bootstrap supports above 0.75 are shown and monophyletic nodes are collapsed. The scale-bar indicates substitutions per site. “*Dermanyssus gallinae*” refers to *Bartonella* sequences obtained from the microbiome of *Dermanyssus gallinae* by Hubert et al. [8]. “Avian and ectoparasite” refers to ASVs from this study

Comparison of the putative *Bartonella 16S* rRNA sequences from our dataset with other *Bartonella* sequences in the literature and homologous sequences from sister taxa showed that all of our sequences are situated in a well-supported clade with the *Bartonella* sequences from previous efforts (Fig. 2). *Mesorhizobium* formed its own clade and *Brucella* and *Ochrobactrum* grouped together in a well-supported clade. One sequence of *Bartonella apis* did not group with the other *Bartonella* sequences and although not a well-supported division, appeared closer to the *Ochrobactrum*/*Brucella* clade. ‘*Bartonella*-like’ sequences isolated from *Dermanyssus gallinae* [8], clearly grouped with our own sequences and appear to form their own clade. There was no clear separation between our putative *Bartonella* strains isolated from bird blood or ectoparasites, with many cases of the same sequence being isolated in both birds and arthropods (Figs. 1, 2).

### *Bartonella* confirmation with *gltA* PCR and sequencing

**Fig. 3:**
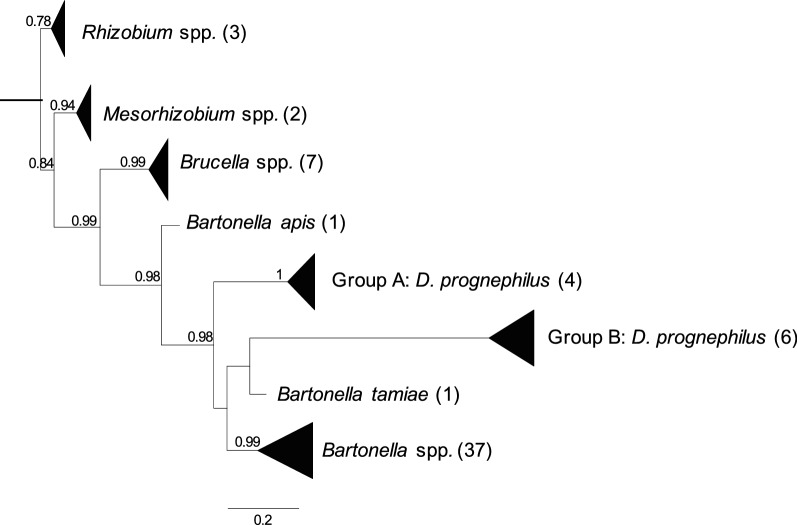
Maximum likelihood phylogenetic tree showing the placement of our *gltA Bartonella* sequences within a wider taxonomy. The tree used the K80 substitution model, optimized invariable sites (I) and optimized across site rate variation (G), with 100 bootstrap replicates and the best of nearest neighbor and subtree pruning with 5 random starts. Numbers on branches show bootstrap support values. Only bootstrap supports above 0.75 are shown and monophyletic nodes are collapsed. The scale-bar indicates substitutions per site. ASVs in ‘Group A’ and ‘Group B’ were all isolated from *Dermanyssus prognephilus* in this study

Phylogenetic analysis showed that our mite *Bartonella gltA* sequences were within a single well-supported clade along with *Bartonella* sequences, supporting the findings from the *16S* rRNA tree. Our sequences were situated in two sub-clades, with the sequences from mites parasitizing purple martins (Group B) being apparently closer to *Bartonella tamiae* and sequences from mites parasitizing tree swallows and eastern bluebirds (Group A) as a sister clade to the other *Bartonella* sequences. *Mesorhizobium* and *Brucella* both formed their own well-supported clades (Fig. 3).

## Discussion

Results from this study have shown that there is a likely *Bartonella* host–vector relationship in an avian system. Our results support the findings of Mascarelli et al. [5] that *Bartonella* occurs in birds and nearly double the list of avian species known to carry *Bartonella*. We also add three species to the pool of avian-associated arthropods known to carry *Bartonella* spp. [6–8, 27]. Our phylogenetic analyses highlight potential expanded diversity within the *Bartonellaceae* associated with avian-system derived *Bartonella* ASVs. Our work implies that we need to broaden our view of the *Bartonellaceae* both in terms of the diversity of its reservoir and vector host range and in terms of phylogenetic diversity within the group.

Our finding of multiple identical *Bartonella* ASVs in birds and arthropods, sometimes within the same nest, strongly suggests a host–vector relationship. All three nest-associated ectoparasites sampled (*Dermanyssus prognephilus, Ceratophyllus idius* and *Protocalliphora sialia*) shared *Bartonella* ASVs with nestling birds and all three species were found in nests continuously throughout the nesting period (HMW, unpublished data), implying that any or all of these species could be vectoring *Bartonella* to the birds. Of the three, *D. prognephilus* may be the most likely vector candidate for two reasons. First, *D. prognephilus* is the most abundant and prevalent nest-associated ectoparasite of all three bird species at our field location, with prevalence approaching 100% for all three species and abundance estimates ranging from means of 300 mites per nest for eastern bluebirds, 600 for tree swallows and almost 2000 for purple martins (HMW, unpublished data). Given a positive correlation between vector abundance and transmission in other host–parasite systems [28–30], *D. prognephilus* may have a significant role in transmission in this system, simply due to the higher encounter rate between birds and mites compared to with the other ectoparasites. Secondly, our microbiome study of *D. prognephilus* suggests a much higher relative abundance of *Bartonella* than in either of the other two ectoparasites considered, or than observed in the birds themselves.

While vector-transmission appears to be a parsimonious explanation of our finding of identical ASVs in birds and ectoparasites, especially given most of the previous evidence that has been gathered for the *Bartonellaceae*, our study does not specifically address transmission modes for the bartonellae. Vector transmission of the *Bartonellaceae* by arthropods has been recorded both directly *via* blood-feeding [1, 31] and indirectly through contamination with the feces of infected arthropods, for example in humans scratching themselves due to infection with the body louse *Pediculus humanus* [32], or when humans acquire cat-scratch fever due to inoculation with infected fleas after cutaneous trauma from an infected cat [2]. In our system of cavity-nesting birds, either mechanism is plausible as birds are not only exposed to bites from the nest-associated ectoparasites, but also to their feces, which accumulate in the nest material.

It is also possible that the three nest-associated ectoparasites studied here are not the only potential *Bartonella* vectors in this system. Other hematophagous arthropods such as chewing lice and biting midges are known to inhabit or visit nests of all three bird species at low abundance and may equally transmit *Bartonella* horizontally to the nestlings. Alternatively, given that *Bartonella* has been recorded as being transmitted maternally in mammals [33], it is also plausible that the nestling birds acquired *Bartonella* vertically. In either of these scenarios, the bacteria could then have been acquired by *D. prognephilus*, *C. idius* and *P. sialia* during blood-feeding. Experimental studies documenting vector competence would be needed to distinguish between these scenarios and to definitively designate vectors and transmission mechanisms in this system. Furthermore, given the relatively short duration of the breeding period in the migratory birds in this study, our study does not capture potential seasonal variation in the prevalence and relative abundance of *Bartonella* in the microbiomes. As previous work has shown a variety of seasonal patterns in *Bartonella* infection status in different systems [34–37], further work could usefully determine whether the patterns in *Bartonella* prevalence and relative abundance observed fluctuate through the seasons.

Our phylogenetic analysis of two genomic regions, supports placement of our sequences within the genus *Bartonella*. However, the sequences do appear to form their own sub-clade within the genus, supported by the close phylogenetic relationship between the *16S* rRNA sequences from this study and those found in the poultry mite, *Dermanyssus gallinae* [8]. It is possible that avian systems are harboring an, as yet, little documented additional source of diversity within the genus *Bartonella* and will lead to a further expansion in our view of the group in the coming years [1, 3].

A growing subset of *Bartonella* species has been associated with human disease [3]. Although we were not able to associate our ASVs with any known species of *Bartonella*, some authors have suggested that any species of *Bartonella* may be capable of human infection [38]. In this case, if *Dermanyssus* mites, in particular, are proven to be a vector of *Bartonella*, there may be consequences of this from a public-health perspective. The *Bartonella* sequences in our samples of *D. prognephilus* group with the sequences obtained in a recent study of the microbiome of the poultry mite *D. gallinae* [8]. Cases of human infestation with *D. gallinae* and associated dermatological lesions are increasing in the medical literature [39, 40] both in poultry workers and in urbanites where infection is associated with infestations from (mainly) pigeon nests on or inside houses and apartment buildings [41]. Furthermore, one study in the Czech Republic reported rashes, fever and tibialgia in a family of people exposed to mites of the *Dermanyssus* genus which were found to be carrying *B. quintana* [7]. Given the possibilities for human contact with these mite species and their demonstrated high relative abundance of *Bartonella* in their microbiomes, further work should examine whether *Dermanyssus* have the potential to vector *Bartonella* to humans and/or wildlife and whether avian-derived *Bartonella* species are pathogenic to humans.

## Conclusions

We have uncovered additional non-mammalian reservoir host species of *Bartonella* and their most likely arthropod vectors. We also presented evidence of expanded diversity within the *Bartonella* genus. Future research may continue to expand our view of the diversity within the *Bartonellaceae* and may seek to confirm the vector relationships suggested here and the potential for avian strains of *Bartonella* to be pathogenic to humans.

## Supplementary information

**Additional file 1: Table S1.** BLASTn analysis of ASVs identified as *Bartonella* spp. in this study.

**Additional file 2: Table S2.** Sequences obtained from GenBank and used in phylogenetic trees.

**Additional file 3: Table S3.** BICc ranking of nucleotide substitution models for the *16S* rRNA tree.

**Additional file 4: Table S4.** BICc ranking of nucleotide substitution models for the *gltA* tree.

**Additional file 5: Table S5.** Showing prevalence of sequences identified to the *Bartonella* genus in *16S* rRNA sequences *via* naïve Bayesian taxonomic classification against the Greengenes database.

## Data Availability

All sequencing data is submitted to and available from NCBI GenBank under the Accession Numbers MN320509–MN320530 and MN3711264–MN371273. All other data in the study are available from the corresponding author upon reasonable requests.

## Abbreviation

ASV: amplicon sequence variant
