# Supplementary material for: Expanding our view of Bartonella and its hosts: Bartonella in nest ectoparasites and their migratory avian hosts
Source: Parasit Vectors. 2020 Jan 10;13:13. doi: 10.1186/s13071-020-3896-7 (PMC6954622; doi:10.1186/s13071-020-3896-7)
Supplement: Supplementary file 5 — Additional file 5: Table S5. Showing prevalence of sequences identified to the Bartonella genus in 16S rRNA sequences via naïve Bayesian taxonomic classification against the Greengenes database. [file 13071_2020_3896_MOESM5_ESM.docx]

**Additional file 5: Table S5.** Showing prevalence of sequences identified to the Bartonella genus in 16S rRNA sequences via naïve Bayesian taxonomic classification against the Greengenes database. Prevalence is shown in each category as the number of samples with Bartonella/total number of samples analyzed.

| **Purple martin (*Progne subis*) nests** | | | | | | | | | | | | |
| --- | --- | --- | --- | --- | --- | --- | --- | --- | --- | --- | --- | --- |
| **Nest ID** | **Nestlings** | | | | ***Dermanyssus prognephilus*** | | | ***Ceratophyllus idius*** | | | | ***Protocalliphora sialia*** |
|  | **16SrRNA** | | | **gltA** | **16SrRNA** | | **gltA** | **16SrRNA** | | | **gltA** | **16srRNA** |
| HQ-C1 | 0/1 | | | NA | 1/1 | | NA | NA | | | NA | 0/1 |
| HQ-C3 | 0/2 | | | NA | 1/1 | | NA | NA | | | NA | NA |
| HQ-C4 | 1/2 | | | NA | 1/1 | | NA | NA | | | NA | NA |
| HQ-G1 | 1/2 | | | NA | NA | | NA | NA | | | NA | NA |
| HQ-G4 | 1/1 | | | 0/1 | 1/1 | | 1/1 | NA | | | NA | NA |
| HQ-G2 | NA | | | 0/1 | 1/1 | | 1/1 | NA | | | NA | 1/2 |
| HQG-G1-b | 1/2 | | | 0/1 | 2/2 | | 1/1 | NA | | | 0/1 | 1/1 |
| HQG-G1-a | 1/1 | | | 0/1 | 4/4 | | 1/1 | NA | | | 0/1 | 0/2 |
| HQG-G2 | NA | | | NA | 4/4 | | NA | NA | | | NA | NA |
| HQG-G4 | 0/1 | | | NA | 1/1 | | NA | NA | | | NA | NA |
| HQG-G5-b | 1/2 | | | 0/1 | 1/1 | | 1/1 | NA | | | 0/1 | NA |
| HQG-G5-a | 1/1 | | | 0/1 | 4/4 | | 1/1 | NA | | | NA | NA |
| HQG-G6 | 2/2 | | | NA | 4/4 | | NA | NA | | | NA | NA |
| HQG-G7 | 1/2 | | | NA | 3/4 | | NA | NA | | | NA | NA |
| HQG-G8 | 2/2 | | | NA |  | | NA | 1/5 | | | NA | NA |
| TP-C10-b | 1/2 | | | NA | 1/1 | | NA | NA | | | NA | NA |
| TP-C10-a | 0/2 | | | NA | 4/4 | | NA | 3/8 | | | NA | NA |
| TP-C11 | 0/1 | | | NA | 1/1 | | NA | NA | | | NA | 1/1 |
| TP-C13 | 0/2 | | | NA | 1/1 | | NA | NA | | | NA |  |
| TP-C3-b | 1/2 | | | NA | 1/1 | | NA | NA | | | NA | 0/2 |
| TP-C3-a | 0/1 | | | NA | NA | | NA | 3/8 | | | NA | 0/2 |
| TP-C4 | 0/2 | | | NA | NA | | NA | 2/8 | | | NA | NA |
| TP-C5-b | 1/2 | | | NA | 1/1 | | NA | 3/4 | | | NA | NA |
| TP-C5-a | 1/1 | | | 0/1 | 4/4 | | 1/1 | 1/4 | | | 0/1 | NA |
| TP-C6-b | 0/1 | | | NA | 1/1 | | NA | 0/4 | | | NA | NA |
| TP-C6-a | 2/2 | | | 0/1 | NA | | 1/1 | 1/4 | | | NA | NA |
| TP-C7-b | 0/2 | | | NA | 3/3 | | NA | 2/3 | | | NA | 0/1 |
| TP-C7-a | 0/2 | | | NA | 2/2 | | NA | 2/2 | | | NA | NA |
| TP-C8 | 1/2 | | | 0/1 | NA | | 0/1 | 1/8 | | | 0/1 | NA |
| TP-C9-b | 0/2 | | | NA | 1/1 | | NA | NA | | | NA | NA |
| TP-C9-a | 0/1 | | | NA | 4/4 | | NA | 2/5 | | | NA | NA |
| TP-C1 | NA | | | NA | 4/4 | | NA | 2/8 | | | NA | NA |
| TP-C2 | NA | | | NA | NA | | NA | 4/8 | | | NA | NA |
| Subtotals | **19/49** | | | **0/9** | **56/57** | | **8/9** | **27/76** | | | **0/5** | **3/12** |
| **Eastern Bluebird (*Sialia sialis*) nests** | | | | | | | | | | | | |
| **Nest ID** | **Nestlings** | | | | ***Dermanyssus prognephilus*** | | | ***Ceratophyllus idius*** | | | | ***Protocalliphora sialia*** |
|  | **16SrRNA** | | **gltA** | | **16SrRNA** | | **gltA** | **16SrRNA** | | **16SrRNA** | | **gltA** |
| EABL-358 | 0/2 | | NA | | NA | | NA | NA | | NA | | NA |
| EABL-385 | 0/2 | | 0/1 | | 2/2 | | 1/1 | NA | | 0/1 | | NA |
| EABL-411 | 1/1 | | NA | | 2/2 | | NA | NA | | NA | | NA |
| EABL-JC1 | 1/1 | | 0/1 | | 1/1 | | 1/1 | NA | | NA | | NA |
| EABL-201 | NA | | NA | | 2/2 | | NA | NA | | NA | | NA |
| Subtotals | **2/6** | | **0/2** | | **7/7** | | **2/2** | **NA** | | **0/1** | | **NA** |
| **Tree Swallow (*Tachycineta bicolor*) nests** | | | | | | | | | | | | |
| **Nest ID** | **Nestlings** | | | | ***Dermanyssus prognephilus*** | | | ***Ceratophyllus idius*** | | | | ***Protocalliphora sialia*** |
|  | **16SrRNA** | **gltA** | | | **16SrRNA** | **gltA** | | **16SrRNA** | **gltA** | | | **16SrRNA** |
| TRES-198 | 1/1 | NA | | | 1/1 | NA | | NA | NA | | | NA |
| TRES-359 | 1/2 | 0/1 | | | 1/1 | 1/1 | | NA | 0/1 | | | NA |
| TRES-361 | 2/2 | 0/1 | | | 1/1 | 1/1 | | NA | 0/1 | | | NA |
| TRES-JC5 | 1/1 | NA | | | 1/1 | NA | | NA | NA | | | NA |
| TRES-363 | NA | NA | | | 1/1 | NA | | NA | NA | | | NA |
| Subtotals | **5/6** | **0/2** | | | **5/5** | **2/2** | | **NA** | **0/2** | | | **NA** |
